# Downregulation of Serine Protease *HTRA1* Is Associated with Poor Survival in Breast Cancer

**DOI:** 10.1371/journal.pone.0060359

**Published:** 2013-04-08

**Authors:** Anna Lehner, Viktor Magdolen, Tibor Schuster, Matthias Kotzsch, Marion Kiechle, Alfons Meindl, Fred C. G. J. Sweep, Paul N. Span, Eva Gross

**Affiliations:** 1 Department of Gynecology and Obstetrics, Technische Universität München, Munich, Germany; 2 Institute of Medical Statistics and Epidemiology, Technische Universität München, Munich, Germany; 3 Institute of Pathology, Dresden University of Technology, Dresden, Germany; 4 Department of Laboratory Medicine, Radboud University Nijmegen Medical Center, Nijmegen, The Netherlands; 5 Department of Radiation Oncology, Radboud University Nijmegen Medical Center, Nijmegen, The Netherlands; University of Barcelona, Spain

## Abstract

HTRA1 is a highly conserved serine protease which has been implicated in suppression of epithelial-to-mesenchymal-transition (EMT) and cell motility in breast cancer. Its prognostic relevance for breast cancer is unclear so far. Therefore, we evaluated the impact of *HTRA1* mRNA expression on patient outcome using a cohort of 131 breast cancer patients as well as a validation cohort including 2809 publically available data sets. Additionally, we aimed at investigating for the presence of promoter hypermethylation as a mechanism for silencing the *HTRA1* gene in breast tumors. *HTRA1* downregulation was detected in more than 50% of the breast cancer specimens and was associated with higher tumor stage (p = 0.025). By applying Cox proportional hazard models, we observed favorable overall (OS) and disease-free survival (DFS) related to high *HTRA1* expression (HR = 0.45 [CI 0.23–0.90], p = 0.023; HR = 0.55 [CI 0.32–0.94], p = 0.028, respectively), with even more pronounced impact in node-positive patients (HR = 0.21 [CI 0.07–0.63], p = 0.006; HR = 0.29 [CI 0.13–0.65], p = 0.002, respectively). Moreover, *HTRA1* remained a statistically significant factor predicting DFS among established clinical parameters in the multivariable analysis. Its impact on patient outcome was independently confirmed in the validation set (for relapse-free survival (n = 2809): HR = 0.79 [CI 0.7–0.9], log-rank p = 0.0003; for OS (n = 971): HR = 0.63 [CI 0.48–0.83], log-rank p = 0.0009). In promoter analyses, we in fact detected methylation of *HTRA1* in a small subset of breast cancer specimens (two out of a series of 12), and in MCF-7 breast cancer cells which exhibited 22-fold lower *HTRA1* mRNA expression levels compared to unmethylated MDA-MB-231 cells. In conclusion, we show that downregulation of *HTRA1* is associated with shorter patient survival, particularly in node-positive breast cancer. Since HTRA1 loss was demonstrated to induce EMT and cancer cell invasion, these patients might benefit from demethylating agents or histone deacetylase inhibitors previously reported to lead to *HTRA1* upregulation, or from novel small-molecule inhibitors targeting EMT-related processes.

## Introduction

The serine protease HTRA1 (Prss11) belongs to the family of high temperature requirement A {HTRA1} proteins. All members of this family consist of a highly conserved protease domain and one or more PDZ domains, exhibiting high structural complexity [1]–[3]. Usually, flat-disk-like trimeric structures (HTRA1) or higher order oligomers (e.g. DegP) are formed. The bacterial homologue DegP appears to have a dual role as a chaperone at normal temperature and as a protease at elevated temperatures [4]. While the physiological function of human HTRA1 remains largely unclear to this end, it was shown to be involved in the pathogenesis of various diseases such as osteoarthritic cartilage [5], [6], preeclampsia [7] or CARASIL (cerebral autosomal recessive arteriopathy with subcortical infarcts and leukoencephalopathy) [8], [9].

Due to its ability to attenuate cell motility [10], growth [11], [12] and invasiveness [11], [13], HTRA1 is also thought to act as a tumor suppressor. Accordingly, downregulation of HTRA1 expression has been reported for various cancer types such as ovarian [12] and endometrial cancer [13], [14] compared to non-malignant tissue. In the breast, HTRA1 expression is prominent in normal ductal glands, whereas its expression is distinctly reduced or even lost in tumor tissues of patients with ductal carcinoma in situ (DCIS) or invasive breast carcinoma [15]. Low HTRA1 expression was found to be associated with poor survival in mesothelioma [16] and hepatocellular carcinoma [17], and has been related to poor response to cytotoxic chemotherapy in ovarian and gastric cancer [18], [19]. He et al. [20] suggested a role for HTRA1 in programmed cell death demonstrating a decrease in X-linked inhibitor of apoptosis protein (XIAP) in ovarian cancer cells dependent on HTRA1 serine protease activity. A proapoptotic function of HTRA1 was also apparent following detachment of epithelial cells. Thus, as a consequence of HTRA1 loss, resistance to anoikis (detachment-induced apoptosis) may contribute to tumor cell dissemination and invasion in metastatic cancer [21].

A variety of substrates such as extracellular matrix proteins are known to be cleaved by secreted HTRA1 [22], [23]. In addition, intracellular HTRA1 was found to co-localize and associate with microtubules through its PDZ domain. Since enhanced expression of HTRA1 attenuated cell motility, whereas HTRA1 loss promoted cell motility, a function of HTRA1 in modulating the stability and dynamics of microtubule assembly has been assumed [10]. Increased motility and invasiveness are also characteristics of epithelial-to-mesenchymal transition (EMT). In breast cancer, HTRA1 loss was in fact accompanied by the acquisition of mesenchymal features as recently shown by Wang et al. [15]. Applying siRNA techniques in the immortalized breast epithelial cell line MCF10A, an inverse correlation of reduced HTRA1 levels with increased expression of mesenchymal markers, higher growth rate and increased migration or invasion was observed [15]. Potentially relevant for anti-cancer therapy, this epithelial-to-mesenchymal transition process also activated ATM and DNA damage response pathways and thus, may further result in poor response to chemotherapy [15].

Taken together, loss of function of HTRA1 may lead to dysregulation of important cellular functions and contribute to tumorigenesis. So far, the basis of HTRA1 downregulation in cancer is unclear, but loss of heterozygosity (LOH) or epigenetic modulations have been postulated as possible mechanisms [12], [15].

Here, we show downregulation of *HTRA1* mRNA expression in a relevant number of breast cancers derived from a cohort of 131 early stage breast cancer patients, validated by public data sets of 2809 cases. To evaluate a possible role of CpG-hypermethylation in causing *HTRA1* downregulation in breast tumors, we subsequently analyzed a set of tumor specimens in addition to breast cancer cell lines by applying bisufite-sequencing techniques.

## Patients and Methods

### Ethics Statement

The study has been approved by the institutional ethical committee of Radboud University Nijmegen Medical Centre, The Netherlands.

### Patients

A series of 131 patients with unilateral, resectable breast cancer, who underwent surgery of their primary tumor between 1986 and 1996, were selected according to the availability of frozen tissue in the tumor bank of the Department of Laboratory Medicine of the Radboud University Nijmegen Medical Centre. This bank contains frozen tumor tissue from patients with breast cancer, obtained from five hospitals of the Comprehensive Cancer Centre East in the Netherlands. After surgical resection of the primary tumor, representative areas of the tumor tissues were selected macroscopically by a pathologist and immediately snap-frozen in liquid nitrogen [24]. Estrogen receptor (ER) and progesterone receptor (PR) levels were measured by a ligand binding assay at the Department of Laboratory Medicine of the Radboud University Nijmegen. Histological grades of the tumors were determined according to Bloom–Richardson criteria, and tumor stage was classified according to the TNM classification system. The clinical data were collected retrospectively. Patients had no previous diagnosis of carcinoma, no distant metastases at time of diagnosis and no evidence of disease within one month after primary surgery. Furthermore, patients receiving neo-adjuvant therapy or with carcinoma in situ only had been excluded from this series. Surgery consisted of modified radical mastectomy for 93 patients (71%) or breast conserving treatment for 38 patients (29%). Postoperative radiotherapy (n = 100; 76.3%) was administered to the breast after incomplete resection, breast conserving treatment, or regional lymph node infiltration. Adjuvant therapy was administered according to guidelines at that time. 61 patients (47%) had received no further treatment. 50 patients (38%) received endocrine therapy, 20 patients (15%) received chemotherapy including three cases in combination with endocrine therapy. Axillary lymph node dissection was carried out in all patients. Lymph node metastasis was observed in 60 (46%) cases. Lymph node involvement was not known in 19 (14.5%) cases. Patient age at diagnosis ranged from 31 to 85 with a median age of 62 years. Follow-up data was available for all patients with the exception of two exact death dates.

### Quantification of *HTRA1* Expression

Total RNA from fresh-frozen breast cancer tissue samples was isolated and reverse-transcribed as published previously [25]. *HTRA1* mRNA expression was determined by TaqMan© real time PCR using the TaqMan Gene expression assay Hs01016151_m1 purchased from Applied Biosystems (Darmstadt, Germany). cDNA was diluted 1∶30 and 3 µl of the diluted cDNA, 15 µl of TaqMan® Universal PCR Master Mix, 1.5 µl TaqMan Gene expression assay and 10.5 µl of H_2_O were pipetted onto a 96well QPCR plate (Peqlab, Erlangen, Germany). The qPCR assays were run on a TaqMan ABI PRISM 7700 Sequence Detection System (Applied Biosystems) according to the manufacturer’s protocol. All samples were measured in duplicates. cDNA of an ovarian carcinoma and a breast cancer sample were included in all runs as calibrator samples. Normalization to human glucose-6-phosphate-dehydrogenase (h-G6PDH) as appropriate housekeeping gene for breast cancer studies was performed as previously described [26]. The ratio between relative *HTRA1* mRNA expression quantities and absolute *h-G6PDH* housekeeping molecule numbers, adjusted to the sample with the lowest *HTRA1* expression, was used for all further calculations and statistical analyses.

### Cell Lines

Breast cancer cell lines MCF-7 (estrogen receptor-positive) and MDA-MB-231 (estrogen receptor-negative) were purchased from American Type Culture Collection (ATCC) (Manassas, VA, USA) and cultured in RPMI 1640 supplemented with penicillin G (100 U/ml), streptomycin (100 mg/ml), L-glutamine and 10% fetal calf serum (Invitrogen, Paisley, UK) at 37°C in a humidified atmosphere containing 5% CO_2_ [27] Cells were routinely checked to be free of mycoplasma. DNA or RNA was extracted from approx. 10^6^ cells which had been harvested in a non-confluent state. DNA was prepared using the Genomic DNA Puregene Purification Kit (Qiagen, Hilden, Germany). For preparation of RNA, the RNeasy kit (Qiagen) was used according to the manufactures protocol for animal cells. cDNA synthesis was performed using the 1^st^ Strand cDNA Synthesis Kit for RT-PCR (AMV) (Roche, Indianapolis, USA) to transcribe 1 µg of RNA each. cDNA was diluted 1∶5 and 1∶20, respectively, and triplicates of each dilution (3 µl) were pipetted onto a 96well QPCR plate (Peqlab, Erlangen, Germany) together with 15 µl of TaqMan® Universal PCR Master Mix and 1.5 µl TaqMan Gene expression assay for *HTRA1* (Hs01016151_m1) or *HPRT* (Hs99999909_m1), respectively, in a final volume of 30 µl. Assays were run in a TaqMan ABI PRISM 7700 Sequence Detection System (Applied Biosystems). *HPRT* was chosen as housekeeping gene for normalization of *HTRA1* expression data in the cell lines. Relative *HTRA1* mRNA expression ratios (calculated from the ratio of *HTRA1* and *HPRT* expression quantities and adjusted to a calibrator sample) were used for further statistical analyses.

### Bisulfite Sequencing

Sodium bisulfite conversion of (un)methylated cytosine was performed using the Epitect Bisulfit kit (Qiagen) and 500 ng of sample DNA. PCR was performed with 3 µl of bisulfite-converted DNA, 0.4 µl AmpliTaq Gold polymerase, 5 µl GeneAmp Buffer 10x with MgCl_2_ (Applied Biosystems), 2 µl MgCl_2_ (25 mM), 5 µl dNTP (2 mM), 2 µl each of forward and reverse Primer (10 pmol/µl) and 30.6 µl H_2_O. Three sets of primers, covering the region −560 to +526 relative to the mRNA start site (Accession No. NG_011554.1), were designed by help of MethPrimer software (www.urogene.org/cgi-bin/methprimer) and are listed in Table S1. Sequencing of the PCR products was carried out on a Genetic Analyser 3130*xl* (Applied Biosystems, Darmstadt, Germany) using Big Dye technology (Applied Biosystems).

The amplified fragments which had shown DNA-methylation in bisufite sequencing analysis, were subcloned using the TOPO-TA cloning kit and One Shot Top10F’ competent cells (Invitrogen, Karlsruhe, Germany). Inserts of clones were sequenced using M13 primers.

### Statistical Analyses

Statistical analyses were carried out using SPSS 17.0 (SPSS Inc. Chicago, IL, USA) and R version 2.11.1 (R Foundation for Statistical Computing, Vienna, Austria). Correlation of relative expression values with clinical/biochemical data were computed with the Spearman-Rho method. Difference in *HTRA1* expression between groups defined by clinical parameters was examined by Mann-Whitney-U-Test or Kruskal-Wallis-Test, depending on the number of compared groups. Overall survival (OS) and disease-free survival (DFS) were considered as long-term endpoints. OS was defined as the time from surgery until death from any cause and DFS was defined as the time from surgery to the first incidence of disease recurrence (local or distant) or death. The Cox proportional hazard model was used to assess univariate and multivariable explanatory ability of the clinical or molecular parameters with respect to OS and DFS. Survival rates were estimated using the Kaplan–Meier method. Differences between survival curves were tested using the logrank test. Ninety-five percent confidence intervals (95% CI) were provided for relevant effect estimates such as hazard ratios (HR). All statistical tests were conducted two-sided and a p-value<0.05 was considered to indicate statistical significance. Optimal cut-off values of quantitative predictive values regarding patient prognosis were obtained with the R-program maxstat.test [28]. This function takes into account the issue of cut-off values derived by multiple testing and computes adjusted p-values.

An online database consisting of gene expression data (Affymetrix HGU133A and HGU133+2 microarrays) and survival information downloaded from GEO was used to validate *HTRA1* expression with respect to the relapse-free survival (RFS) and OS in 2809 and 971 breast cancer patients, respectively. Distant-metastasis-free survival (DMFS) was analyzed in 311 patients. Version 2012 was used (last update 2013.02.26) applying a follow-up time of 15 years (see ref. [29]). The Affymetrix-ID of the HTRA1 probe is 201185_at.

This study adheres to the REMARK criteria for tumor marker studies [30].

## Results

### Patient Characteristics

A cohort of 131 patients with unilateral breast cancer was collected for this study. Clinical data are listed in Table 1. The median follow-up time was 92.8 months, ranging from 3 to 169 months. Recurrence or death was observed in 38% (50 out of 131) and 29% (37 out of 129) of the cases, respectively (combined events of recurrence and/or death encompassed 58 (44%) cases). The Kaplan Meier estimates for the 5- and 10-year overall survival (OS) rates in the entire patient cohort were 85% (±3,2% standard error SE) and 66% (±4.9%), respectively. 5- and 10-year recurrence-free times were obtained in 75% (±4.0%) and 54.5% (±5.2%) of the patients, respectively. Combined disease-free survival rates (DFS, neither death nor recurrence) were estimated to 71.5% (±4.0%) for 5 years and to 50% (±5.0%) for 10 years. For the lymph node-positive subgroup, the following outcome rates were observed: OS: 82% (±5.1%) for 5 years and 68% (±6.8%) for 10 years; DFS: 68% (±6.1%) for 5 years and 46% (±7.4%) for 10 years.

**Table 1:** *HTRA1* mRNA expression levels in a cohort of 131 breast cancer patients.

Variable	N = 131^a^	*HTRA1* Expression Median^b^ (IR^c^)	*p*
**Age**			0.271^d^
<50	23	48 (54)	
>50	108	37 (51)	
**Menopausal status**			0.337^d^
pre−/perimenopausal	28	45 (47)	
postmenopausal	103	37 (56)	
**Lymph node status**			0.439^e^
negative	52	41 (47)	
1–3 lymph nodes	43	48 (63)	
4–9 lymph nodes	11	34 (41)	
>9 lymph nodes	6	19 (45)	
**Tumor stage (pT)**			**0.025** e
1	39	53 (58)	
2	72	40 (49)	
3+4	18	20 (26)	
**Grading**			0.587^d^
1+2	49	45 (60)	
3	46	30 (49)	
**Estrogen receptor**			0.672^d^
negative	37	32 (65)	
positive	91	43 (54)	
**Progesterone receptor**			0.219^d^
negative	51	31 (56)	
positive	77	45 (56)	
**Surgery**			0.320^d^
breast conserving	38	47 (60)	
mastectomy	93	32 (49)	

a Due to missing data the sum of values may be lower than 131.

b Median of relative *HTRA1* mRNA expression values after normalization to glucose-6-phosphate-dehydrogenase (h-G6PDH) expression and adjustment to the sample with lowest *HTRA1* expression.

c IR: interquartile range.

d Mann-Whitney-U test.

e Kruskal-Wallis test.

### *HTRA1* Expression in Groups Defined by Clinicopathologic Parameters

Relative *HTRA1* mRNA expression ratios in the breast cancer specimens ranged from 1 to 308-fold compared to the sample exhibiting the lowest *HTRA1* expression, the median expression level was 38. Comparison of the expression data between patient groups defined by clinical and histomorphological parameters (Table 1) revealed a statistical significant difference only for pT categories, indicating a decrease in *HTRA1* mRNA levels with increasing tumor stage (p = 0.025). Interestingly, *HTRA1* expression levels did not exceed relative values higher than 55 in the presence of very high ER concentrations >400 fmol/mg protein.

### *HTRA1* Expression and Patient Outcome

**Figure 1:**
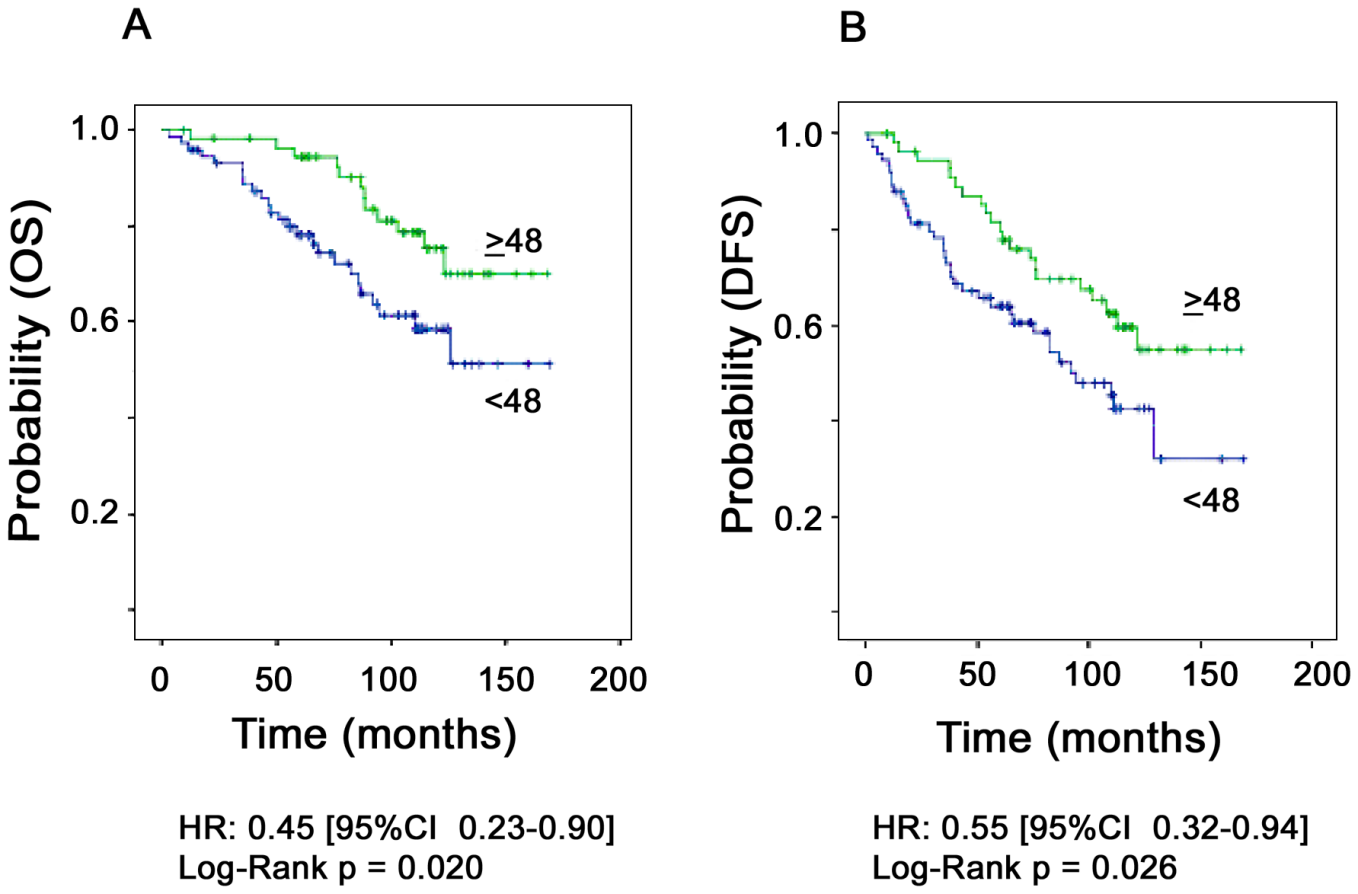
Patient outcome as a function of *HTRA1* mRNA expression in breast cancer patients. **A**. Overall survival (n = 129). **B**. Disease-free survival (n = 131).

We next assessed the impact of *HTRA1* mRNA expression on patient survival using OS and DFS as outcome variables. With respect to an optimized cut-off value of ≥48, deduced by means of the program R (Figure S1), 56 (43%) tumor specimens showed high *HTRA1* expression and 75 (57%) low expression. High *HTRA1* mRNA expression levels were found to be associated with favorable OS and DFS (Figure 1A and B), showing a significantly reduced risk for recurrent disease and/or death in the entire patient cohort (HR = 0.45 [CI 0.23–0.90], p = 0.023 for OS; HR = 0.55 [CI 0.32–0.94], p = 0.028 for DFS; Table 2 and 3). Moreover, *HTRA1* expression was maintained as a statistically significant factor which predicted outcome (DFS) independent from nodal status when tested among the established clinical factors age, tumor stage, nodal involvement and nuclear grading (binary variables) in the multivariable analysis (Table 4).

**Table 2:** Univariate Cox proportional hazard ratios for OS with respect to clinical parameters and *HTRA1* mRNA expression levels.

Variable	N = 129	Numberof events	Hazard Ratio (95% CI)	*p* a
***HTRA1*** ** expression**				
low	73	25	1	**0.023**
high	56	12	0.45 (0.23–0.90)	
**Age**				
<50	23	8	1	0.444
>50	106	29	0.74 (0.34–1.61)	
**Menopausal status**				
Pre-/peri-	28	10	1	0.276
postmenopausal	101	27	0.82 (0.57–1.18)	
**Lymph node status**				
negative	51	13	1	**0.005**
1–3 lymph nodes	43	10	0.90 (0.40–2.07)	
4–9 lymph nodes	11	3	1.41 (0.40–4.95)	
>9 lymph nodes	6	5	5.77 (2.02–16.51)	
unknown	18			
**Tumor stage (pT)**				
1	39	10	1	0.308
2	71	20	1.19 (0.56–2.55)	
3+4	17	7	2.09 (0.79–5.52)	
unknown	2			
**Nuclear grading**				
1+2	49	15	1	0.896
3	45	13	0.95 (0.45–2.00)	
unknown	35			
**Estrogen receptor**				
negative	35	8	1	0.387
positive	91	28	1.42 (0.64–3.13)	
unknown	3			
**Progesterone receptor**				
negative	49	12	1	0.435
positive	77	24	1.32 (0.66–2.64)	
unknown	3			
**Adjuvant therapy**				
none	60	19	1	0.850
endocrine only	49	13	1.07 (0.40–2.87)	
chemotherapy	20	5	0.87 (0.31–2.45)	

a Univariate Cox regression analysis; 95% CI, 95% confidence interval; OS, overall survival with endpoint death of any cause.

**Table 3:** Univariate Cox proportional hazard ratios for DFS with respect to clinical parameters and *HTRA1* mRNA expression levels.

Variable	N = 131	Numberof events	Hazard Ratio (95% CI)	*p* a
***HTRA1*** ** expression**				
low	75	37	1	**0.028**
high	56	21	0.55 (0.32–0.94)	
**Age**				
<50	23	11	1	0.329
>50	108	47	0.72 (0.37–1.39)	
**Menopausal status**				
Pre-/peri-	28	14	1	0.200
postmenopausal	103	44	0.82 (0.61–1.11)	
**Lymph node status**				
negative	52	20	1	**0.023**
1–3 lymph nodes	43	20	1.22 (0.64–2.27)	
4–9 lymph nodes	11	4	1.19 (0.41–3.47)	
>9 lymph nodes	6	5	4.82 (1.77–13.14)	
unknown	19			
**Tumor stage (pT)**				
1	39	16	1	0.148
2	72	31	1.18 (0.65–2.17)	
3+4	18	11	2.26 (0.97–4.55)	
unknown	2			
**Nuclear grading**				
1+2	49	22	1	0.489
3	46	23	1.23 (0.69–2.21)	
unknown	36			
**Estrogen receptor**				
negative	37	17	1	0.983
positive	91	40	1.01 (0.57–1.78)	
unknown	3			
**Progesterone receptor**				
negative	51	21	1	0.443
positive	77	36	1.24 (0.72–2.12)	
unknown	3			
**Adjuvant therapy**				
none	61	25	1	0.730
endocrine only	50	23	1.01 (0.56–1.86)	
chemotherapy	20	10	1.36 (0.63–2.92)	

a Univariate Cox regression analysis; 95% CI, 95% confidence interval; DFS, disease-free survival with endpoints recurrence and/or death.

**Table 4:** Multivariable Cox regression analysis for DFS.

	Univariate Cox-Regression		Multivariable Cox-Regression	
Variable	HR (95% CI)	*p*	HR (95% CI)	*p*
*HTRA1*expression (low/high)	0.55 (0.32–0.94)	**0.028**	0.46 (0.23–0.92)	**0.028**
Nodal status (negative/positive)	1.39 (0.79–2.46)	0.256	2.12 (1.07–4.22)	**0.032**
Tumor stage (pT1+2/pT3+4)	2.11 (1.09–4.09)	**0.027**	1.29 (0.50–3.33)	0.597
Age at diagnosis (<50 ys/>50 ys)	0.72 (0.37–1.39)	0.329	0.54 (0.25–1.17)	0.119
Nuclear grading (G1+2/G3)	1.23 (0.69–2.21)	0.489	1.21 (0.59–2.51)	0.606

Number of patients in multivariable analysis: n = 80; number of events of recurrence and/or death in multivariable analysis: 38.

For both analyses, binary variables are used; HR, hazard ratio; 95% CI, 95% confidence interval.

DFS: disease-free survival with endpoints recurrence and/or death.

### Validation Set

Public data sets of breast cancer patients derived from GEO expression data were used for validation [29]. We could confirm a statistically significant effect of high *HTRA1* mRNA expression (based on Affymetrix HGU133A and HGU133+2 microarrays) on patient outcome: In 2809 patients with 15-year-follow up, a HR = 0.79 [CI 0.7–0.9], log-rank p = 0.0003, was defined for the relapse-free survival (RFS). The data set available for the 15-year-OS included 971 patients and yielded a HR = 0.63 [CI 0.48–0.83], log-rank p = 0.0009 (Figure S2).

### Subgroup Analysis

**Figure 2:**
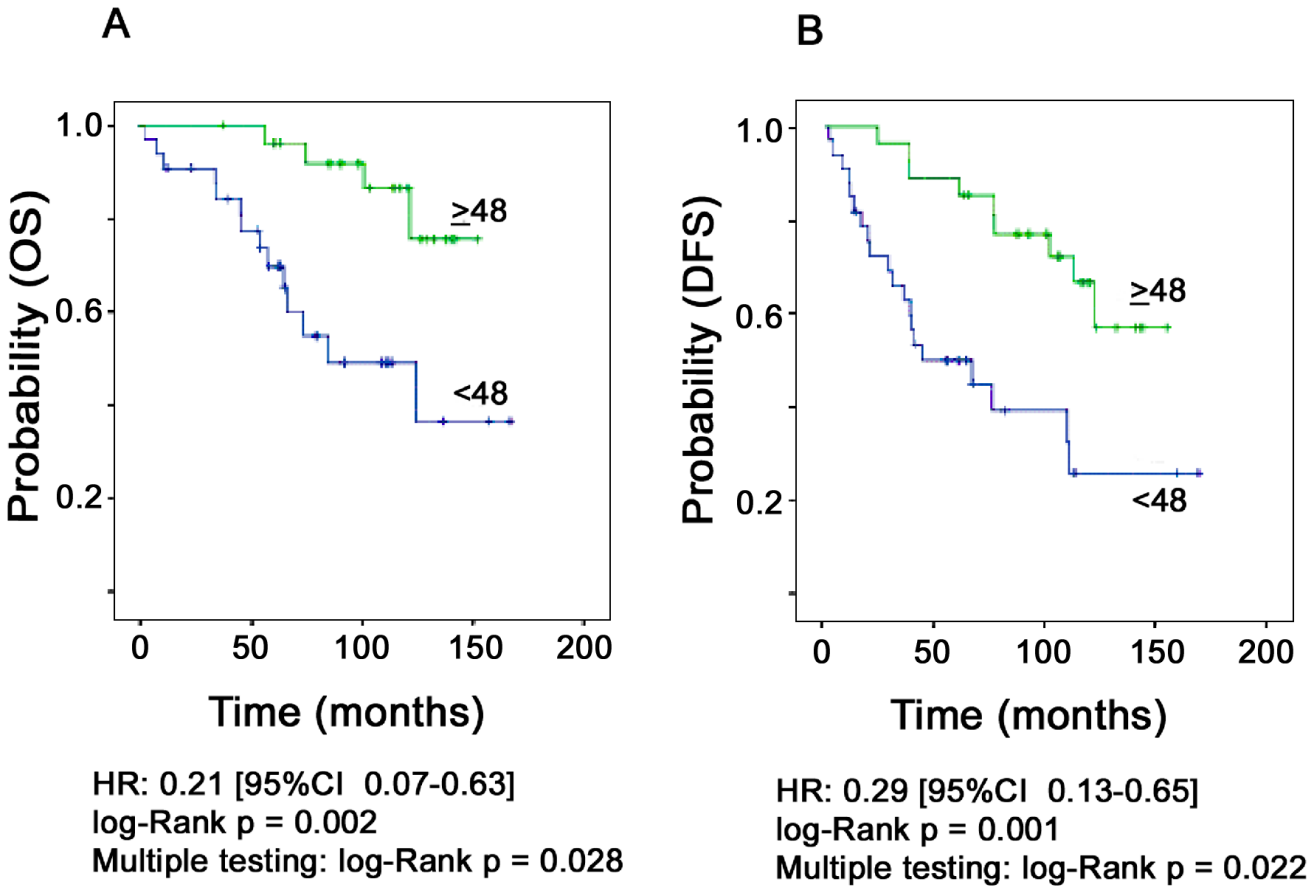
Patient outcome in node-positive breast cancer patients as a function of *HTRA1* mRNA expression. **A**. Overall survival (n = 60). **B**. Disease-free survival (n = 60). Multiple testing performed with the R-package maxstat.test [28] is provided.

Stratification of patients by clinicopathological parameters revealed a more pronounced impact of *HTRA1* mRNA expression in the node-positive subgroup of our patient cohort (n = 60). We observed a considerable lower risk for death (5-fold) or disease progression (3-fold) with higher *HTRA1* concentrations: HR = 0.21 [CI 0.07–0.63], p = 0.006 for OS; HR = 0.29 [CI 0.13–0.65], p = 0.002 for DFS (Figure 2A and B). In the multivariable model including tumor stage and adjuvant treatment, *HTRA1* expression was confirmed as a clinically relevant parameter predicting OS or DFS (Table 5 and 6). Adjusted for therapy mode (none, endocrine, chemotherapy), the impact of *HTRA1* expression remained statistically significant as well: OS: HR (*HTRA1*) = 0.23 [CI 0.07–0.72]; p = 0.012; DFS: HR (*HTRA1*) = 0.29 [CI 0.13–0.67]; p = 0.004.

**Table 5:** Multivariable Cox regression analysis* for the risk of death (OS) in node-positive breast cancer patients.

	Univariate Cox-Regression		Multivariable Cox-Regression	
Variable	HR (95% CI)	*p*	HR (95% CI)	*p*
*HTRA1*expression (low/high)	0.21 (0.07–0.63)	**0.006**	0.25 (0.08–0.80)	**0.020**
Tumor stage (pT1+2/pT3+4)	2.51 (0.93–6.78)	0.070	1.44 (0.48–4.31)	0.511
Adjuvant therapy (no/yes)	0.33 (0.12–0.928)	**0.036**	0.54 (0.18–1.64)	0.277

* Number of patients in multivariable analysis: n = 60; number of events of death: 18; binary variables are used; HR, hazard ratio; 95% CI, 95% confidence interval; OS, overall survival with endpoint death of any cause.

**Table 6:** Multivariable Cox regression analysis* for DFS in node-positive breast cancer patients.

	Univariate Cox-Regression		Multivariable Cox-Regression	
Variable	HR (95% CI)	*p*	HR (95% CI)	*p*
*HTRA1*expression (low/high)	0.29 (0.13–0.65)	**0.002**	0.34 (0.15–0.79)	**0.012**
Tumor stage (pT1+2/pT3+4)	2.56 (1.15–5.71)	**0.021**	1.81 (0.79–4.18)	0.162
Adjuvant therapy (no/yes)	0.47 (0.18–1.24)	0.127	0.70 (0.26–1.90)	0.480

* Number of patients in multivariable analysis: n = 60; number of events of recurrence and/or death: 29; binary variables are used; HR, hazard ratio; 95% CI, 95% confidence interval; DFS, disease-free survival with endpoints recurrence and/or death.

On the other hand, no positive effect of *HTRA1* expression was apparent in mostly untreated node-negative patients (HR = 1.24 [CI 0.52–2.98], p = 0.663 for DFS). Since 53 out of our 60 patients with node-positive disease had received endocrine (n = 35) and/or chemotherapy (n = 18), the observed effects of *HTRA1* appear to be largely related to the patient subgroup which is adjuvantly treated. These results are also reflected by the publically available data set which showed greater benefit from endocrine therapy (n = 743) at high *HTRA1* expression (HR = 0.66 [CI 0.5–0.89], log-rank p = 0.006 for RFS), while *HTRA1* expression had no statistically significant impact on RFS in 933 systemically untreated patients (HR = 0.84 [CI 0.68–1.05], log-rank p = 0.123).

### Methylation Analysis

**Figure 3:**
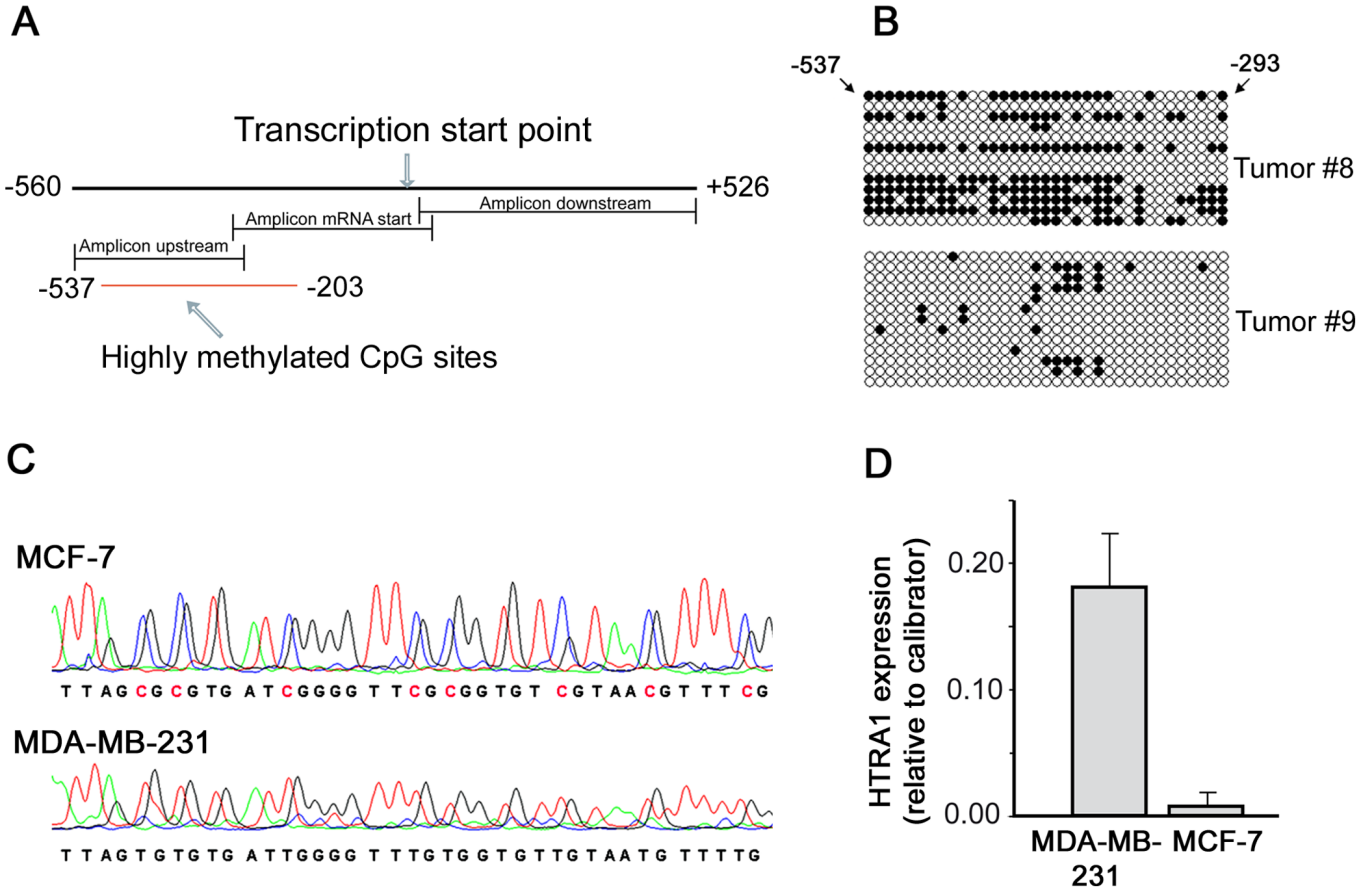
Methlylation analysis of the *HTRA1* promoter. **A**. Schematic illustration of the studied region covering nucleotides –560 to +526 relative to the transcription start site. The location of amplicons and of highly methylated CpG sites determined in this study is indicated. **B**. Methylation status of tumor samples #8 and #9: Potential CpG sites and the results of 13 analyzed clones in the amplicon upstream of the transcription start site are shown (black circles define methylated sites). The most distant CpG site is located at position −537, the most proximal site at position −293. **C**. Electropherograms obtained by genomic bisulfite sequencing of MCF-7 und MDA-MB-231 breast cancer cell lines. MCF-7 cells showed strong methylation of all CpG islands in the “upstream” region (shown here in part) and, as the only sample, in the first seven CpG-islands in the PCR fragment “mRNA start”. Methylated CpG sites are highlighted. No significant methylation was observed in MDA-MB-231 cells. **D**. Quantification of *HTRA1* mRNA expression in two breast cancer cell lines. Relative *HTRA1* expression levels, normalized to *HPRT* and adjusted to an ovarian cancer sample as calibrator, are shown in MCF-7 and MDA-MB-231 cells. SD values of two independent experiments are indicated. Mean difference in expression between both cell lines was 22-fold.

To investigate *HTRA1* promoter hypermethylation as a possible mechanism of *HTRA1* downregulation in breast tumors, we analyzed the extent of CpG methylation in a region of approx. 1000 bp including the *HTRA1* transcription start point as illustrated in Figure 3A. Two sets of six tumor samples displaying high and low expression of *HTRA1*, respectively, were chosen from breast cancer specimens of our study. Additionally, we analyzed two breast cancer cell lines showing different *HTRA1* expression levels. Relevant promoter methylation was detected only in two out of the 12 tumor specimens, exhibiting low relative *HTRA1* expression levels of 2.5 and 2.7, and in the MCF-7 cell line (Figure 3B and C). Subcloning of the tumor-derived amplicons (tumors #8 and #9) revealed DNA methylation in these tumor specimens within a region of nt −537 to −293 upstream of the mRNA start point. Patients #8 and #9 both showed disease recurrence after 75 and 34 months, respectively; Patient #8 died of disease. Almost complete methylation was observed in MCF-7 cells within a stretch of 43 potential CpG sites (position –537 to −203 relative to mRNA start point). Compared with these cells, MDA-MB-231 breast cancer cells displayed no detectable methylation accompanied by a 22-fold higher *HTRA1* mRNA expression (Figure 3C and D). According to the data of Wang et al. [15], treatment with demethylating agents does not further increase *HTRA1* transcripts in MDA-MB-231 cells. Thus, it is reasonable that all relevant CpG sites have been examined and found unaffected in this cell line.

## Discussion

Numerous studies revealed downregulation of the serine protease HTRA1 in cancer. In particular, studies in ovarian and endometrial cancer reported reduced HTRA1 protein levels in 59% [12] and 57% [13] of the cases. In these tumors, absence of HTRA1 expression has also been associated with more aggressive tumor phenotypes and higher grading. HTRA1 expression was also reduced or entirely lost in six studied breast cancer tissues and five human breast cancer cell lines as reported by Wang et al. [15]. In the present study, we have investigated the presence of *HTRA1* transcripts in a panel of 131 breast cancer specimens by qPCR. Our patient cohort displayed a wide range of relative *HTRA1* mRNA expression levels. Lower *HTRA1* mRNA values were indeed observed in patients exhibiting more aggressive clinical characteristics like high grading or high lymph node infiltration (≥4 lymph nodes), however, a statistically significant association was obtained only between low *HTRA1* mRNA expression and higher tumor stage (see Table 1).

Evaluating the impact of *HTRA1* expression on breast cancer outcome, we could show favorable survival (OS and DFS) in relation to high *HTRA1* mRNA expression. Moreover, *HTRA1* revealed to be a survival-related factor providing independent prognostic information in the multivariable model. We subsequently validated our data using publically available data sets based on Affymetrix HGU133A and HGU133+2 microarrays [29], which provided relapse-free survival (RFS) data of 2809 breast cancer patients and OS data of 971 patients within a follow up time of at least 15 years. Consistent with our results, the validation set showed better patient survival associated with high *HTRA1* mRNA expression. Taking into account the relative heterogeneous nature of this panel of up to 2809 breast cancer cases, the impact of *HTRA1* was less pronounced (HR = 0.79 for RFS), but high statistical significance was obtained (log-rank p = 0.0003). Best cut-off points in this analysis were slightly above the median *HTRA1* expression level, compatible with our calculated optimized cut-off value. Thus, *HTRA1* mRNA expression appears as a robust marker for breast cancer outcome supported by two different methodologies to assess transcript levels. Furthermore, correlation of *HTRA1* mRNA and protein expression has been reported for a number of cancers such as endometrial and ovarian cancer as well as for melanoma cell lines [11], [12], [31], suggesting equal relevance of mRNA compared to protein measurement. This is also supported by coincident downregulation of mRNA and protein expression levels of HTRA1 in Syrian hamster kidney after prolonged estrogenization [32].

In subgroup analyses, we observed the most pronounced effect of HTRA1 proficiency in node-positive breast cancer. It might be reasonable to assume a higher relevance of *HTRA1* expression especially in breast cancer patients with lymph node involvement, because these patients usually receive adjuvant therapy due to their greater risk of disease progression [33]. Accordingly, we demonstrated that 88% of our node-positive patients had been treated with endocrine and/or chemotherapy, whereas only three out of our 52 node-negative patients were adjuvantly treated. Hence, together with our data obtained in the validation set, these data may support previous results in gastric and ovarian cancer [18], [19] which have linked HTRA1 proficiency to better therapeutic responsiveness indicating that HTRA1 is a predictive marker. Similarly, in a breast cancer study, HTRA1 was one among a panel of three markers which predicted response to doxorubicin-based chemotherapy [34]. In contrast, low HTRA1 expression was previously shown to trigger EMT in breast cancer cells [15] which is most likely involved in drug resistance [35]. Furthermore, low HTRA1 expression appears to be associated with more aggressive clinical characteristics. In our breast cancer patient cohort, we observed reduced *HTRA1* expression levels particularly in patients exhibiting unfavorable clinical features such as high numbers of affected lymph nodes (≥4 lymph nodes; see Table 1). Because an even greater HTRA1 downregulation in lymph node metastases compared to the primary sites was evident in lung cancer [36] and malignant melanoma [11], this strongly points to a particular benefit for node-positive patients to have high expression of the tumor suppressor HTRA1.

Interestingly, GEO-data-derived results computed by us for the 10-year-distant-metastasis-free survival (DMFS) in untreated patients may also point to a “truly” prognostic value of *HTRA1* expression regarding the risk of metastasis. By analyzing the “truly prognostic data set” (n = 311) we found strong association of high *HTRA1* mRNA expression levels with longer DMFS showing a HR = 0.45 [CI 0.31–0.65], log-rank p = 0.0000097 (see Figure S3). The lower risk of metastasis at high *HTRA1* expression levels is most likely related to the anti-migratory [7], [10], [21] and proapoptotic functions [12], [20] described for this serine protease. In particular, HTRA1 downregulation has been previously shown to be associated with the metastatic phenotype of melanoma cells, while HTRA1 expression suppressed growth and matrix invasion of metastatic cells [11]. Furthermore, stimulation of cancer cell migration and invasion following HTRA1 inhibition could be demonstrated in SKOV3 cells [10] and in immortalized breast epithelial cells [15]. Both studies used siRNA techniques to knock down HTRA1 expression, while forced HTRA1 expression attenuated cell migration. A mouse model strongly supports these data as increased numbers of micrometastases could be found in the lung of mice after i.v. injection of endometrial cancer cells expressing HTRA1-siRNA [13].

A particular feature of metastatic breast cancer is activation of EMT which is known to promote growth, motility and invasion. Downregulation of HTRA1 was indeed shown to stimulate expression of mesenchymal markers and characteristics in breast cancer cells [15]. HTRA1 was also shown to regulate TGF-ß signaling. Decrease of HTRA1′s proteolytic activity, e.g. in CARASIL [9], leads to increased extracellular levels of TGF-ß. Since TGF-ß is a potent inducer of EMT [37], [38], this would speak in favor of a direct role of HTRA1 in controlling active TGF-ß levels, thereby suppressing EMT. Targeting EMT-related processes downstream of HTRA1 might therefore proof an attractive new strategy in the treatment of breast cancer. In this context, Fang et al. very recently introduced a novel inhibitor of TGF-ß receptor 1, YR-290, which could be demonstrated to markedly block TGF-ß-mediated EMT and breast cancer cell invasion [39].

Alternatively, new therapeutic strategies may exploit mechanisms to stimulate re-expression of HTRA1, although the basis of *HTRA1* downregulation in cancer cells might be complex. Overstimulation of the estrogen pathway may contribute to a decrease in HTRA1 expression as described in a model of Syrian hamster nephrocarcinogenesis after prolonged estrogenization [32]. Similarly, we did not see any cases with pronounced *HTRA1* mRNA expression in breast cancer samples displaying very high estrogen receptor values. Epigenetic events have been postulated as other potential mechanisms to reduce *HTRA1* expression levels in cancer. In particular, Chien et al [12] reported upregulation of *HTRA1* by treatment of ovarian cancer cells with the demethylating agent deaza-cytidin. Consistent with these findings, promoter methylation within the *HTRA1* gene has been very recently demonstrated in four out of five breast cancer cell lines [15]. Independently, we have screened a larger region of approx. 1000 bp including the transcription start point for the presence of *HTRA1* methylation. Analyzing the two breast cancer cell lines MCF-7 and MDA-MB-231, we could define 43 potential CpG sites (position –537 to −203 relative to mRNA start) which were found to be almost fully methylated in MCF-7 cells. In concordance with the data of Wang et al. [15], we did not detect significant methylation in this region in MDA-MB 231 cells exhibiting >20-fold higher *HTRA1* mRNA expression compared to the MCF-7 cell line. These data suggest that the studied promoter region upstream of the transcription start point is apparently important for *HTRA1* expression. Remarkably, also two out of six low *HTRA1* -expressing tumor samples derived from our breast cancer cohort showed methylation in a smaller, distal stretch of this particular region (recent data obtained by Wang et al. with the MDA-MB-468 cell line suggested that the more distal CpG sites might in fact be the most relevant ones for HTRA1 expression [15]). None of the six studied high-expressing tumors indicated relevant *HTRA1* promoter methylation. However, as only one third of the tumors with *HTRA1* downregulation seemed to show methylation events, such events are likely to represent only one epigenetic mechanism for regulating HTRA1 expression. Indeed, *HTRA1/Prss11* has been previously found to be a target for histone deacetylase 1 (HDAC1) in mouse and *HTRA1/Prss11* upregulation was reported by HDAC inhibition [40]. Concordant with these results, Wang et al. [15] demonstrated upregulation of *HTRA1* mRNA in methylation-negative MDA-MB-231 cells by HDAC inhibitors, while demethylating agents resulted in increased *HTRA1* expression in highly methylated M4A4 cells. Thus, HTRA1 downregulation in cancer might in fact be explained by epigenetic mechanisms such as promoter methylation or histone-deacetylation [15].

Further investigation will be necessary to unravel the regulation of HTRA1 expression in breast cancer in order to stimulate re-expression of this tumor-suppressor for the purpose of clinical intervention, e.g, by HDAC inhibitors or demethylating agents. With respect to the role of HTRA1 in EMT suppression, breast cancer patients with low HTRA1 expression might also be suited for drugs targeting EMT-related processes via inhibition of TGF-ß signaling [39].

## Supporting Information

Figure S1 **Determination of the optimal cut-off value for quantitative** ***HtrA1*** **mRNA expression levels.** The best cut.off value with respect to patient outcome was obtained with the R-program maxstat.test [28].
(TIF)

Figure S2 ***HTRA1*** **expression and patient outcome in the validation set.** An online database consisting of gene expression data and survival information downloaded from GEO (Affymetrix HGU133A and HGU133+2 microarrays) was used for correlation with outcome within a period of 15 years [29]. **A.** Relapse-free survival in 2809 breast cancer patients. Median *HTRA1* expression was 3979. Auto-selected best cut-off used in analysis was 4417. **B.** Overall survival in 971 breast cancer patients. Auto-selected best cut-off used in analysis was 5190.
(PPT)

Figure S3 ***HTRA1*** **expression and distant metastasis-free survival in the truly prognostic data set.** An online database consisting of gene expression data and survival information downloaded from GEO (Affymetrix HGU133A and HGU133+2 microarrays) was used for correlation with distant metastasis-free survival [29]. Survival data of 311 systemically untreated breast cancer patients for up to 10 years were calculated. Auto-selected best cut-off used in analysis was 3366.
(PPT)

Table S1 **Primer set for bisulfite sequencing.**
(DOC)
