# Supplementary figures and images for: Downregulation of Serine Protease HTRA1 Is Associated with Poor Survival in Breast Cancer
Source: PLoS One. 2013 Apr 8;8(4):e60359. doi: 10.1371/journal.pone.0060359 (PMC3620283; doi:10.1371/journal.pone.0060359)

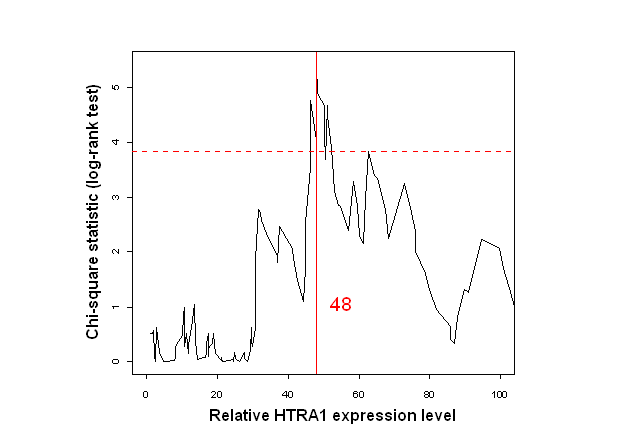

Supplement: Figure S1 — Determination of the optimal cut-off value for quantitative HtrA1 mRNA expression levels. The best cut.off value with respect to patient outcome was obtained with the R-program maxstat.test [28]. (TIF) [file pone.0060359.s001.tif]

## Slide 1
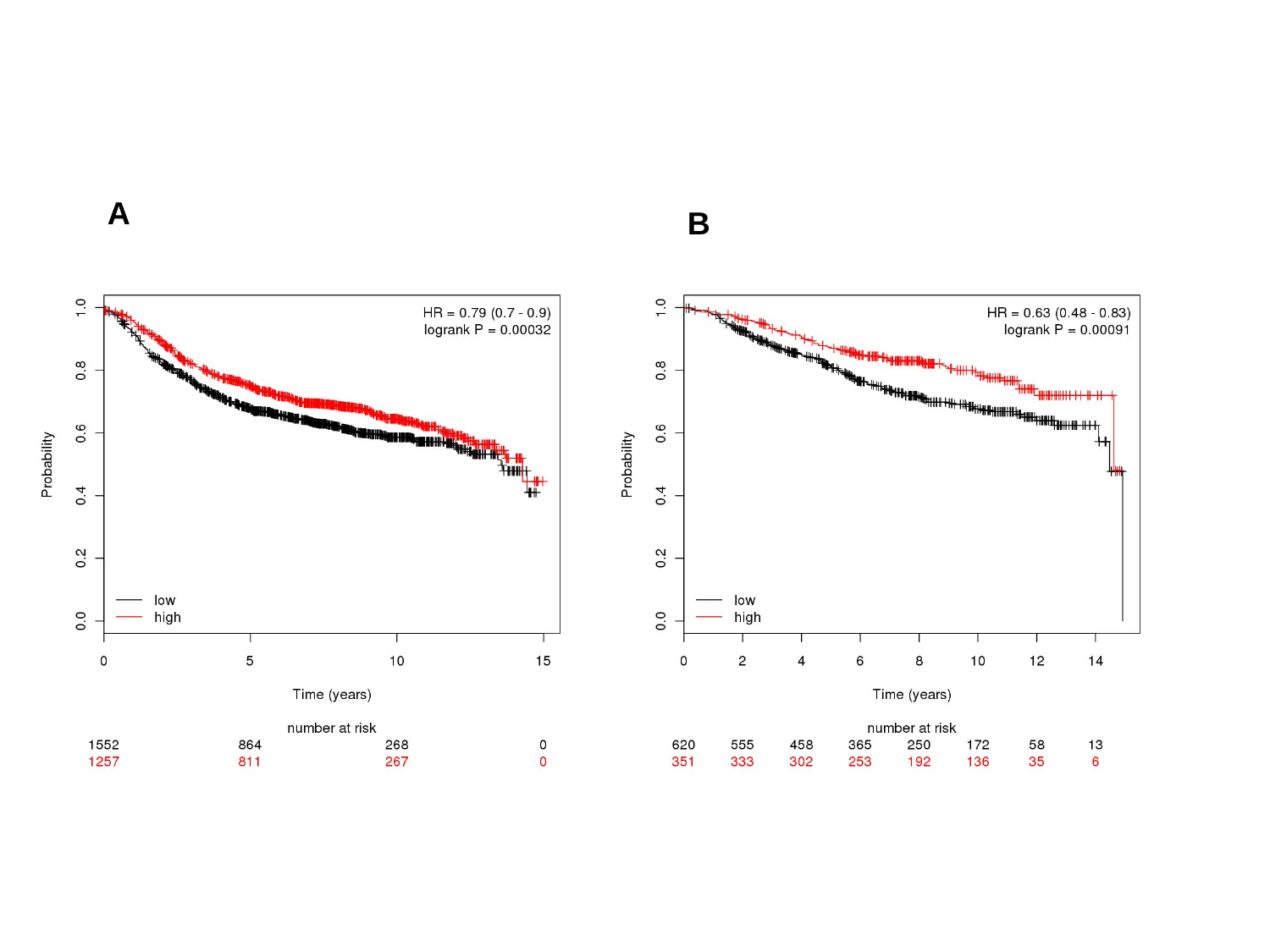

A
B

Supplement: Figure S2 — HTRA1 expression and patient outcome in the validation set. An online database consisting of gene expression data and survival information downloaded from GEO (Affymetrix HGU133A and HGU133+2 microarrays) was used for correlation with outcome within a period of 15 years [29]. A. Relapse-free survival in 2809 breast cancer patients. Median HTRA1 expression was 3979. Auto-selected best cut-off used in analysis was 4417. B. Overall survival in 971 breast cancer patients. Auto-selected best cut-off used in analysis was 5190. (PPT) [file pone.0060359.s002.ppt]

## Slide 1
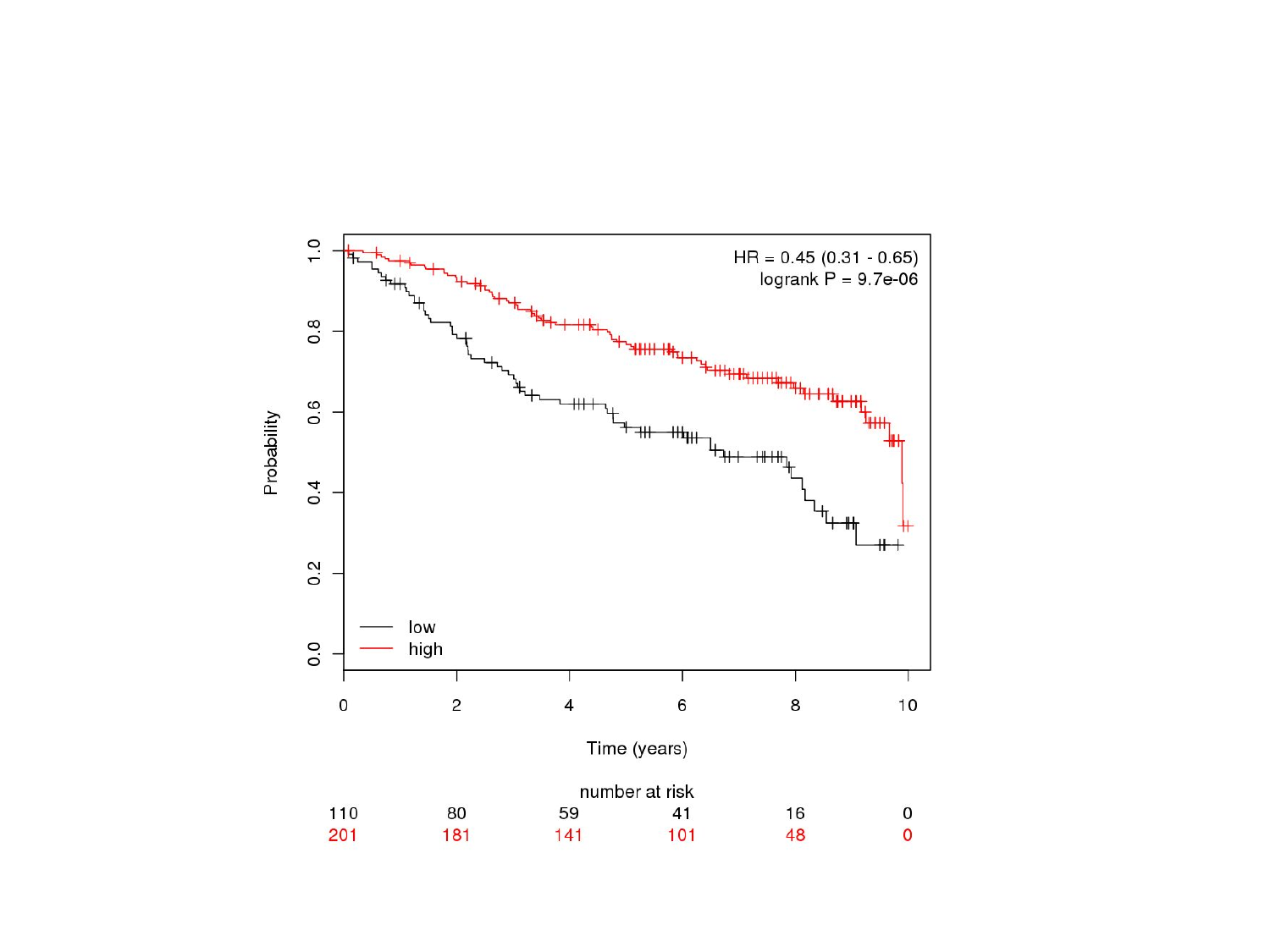

Supplement: Figure S3 — HTRA1 expression and distant metastasis-free survival in the truly prognostic data set. An online database consisting of gene expression data and survival information downloaded from GEO (Affymetrix HGU133A and HGU133+2 microarrays) was used for correlation with distant metastasis-free survival [29]. Survival data of 311 systemically untreated breast cancer patients for up to 10 years were calculated. Auto-selected best cut-off used in analysis was 3366. (PPT) [file pone.0060359.s003.ppt]
