# Supplementary material for: Downregulation of Serine Protease HTRA1 Is Associated with Poor Survival in Breast Cancer
Source: PLoS One. 2013 Apr 8;8(4):e60359. doi: 10.1371/journal.pone.0060359 (PMC3620283; doi:10.1371/journal.pone.0060359)
Supplement: Table S1 — Primer set for bisulfite sequencing. (DOC) [file pone.0060359.s004.doc]

Supporting Information Table S1: Primer set for bisulfite sequencing

| **Region** | **Primer** | **Sequence** | **Length [bp]** |
| --- | --- | --- | --- |
| Upstream | forward | 5’-TAT TAT TTT ATT GTG GGT TTG GG-3’ | 305 |
|  | reverse | 5’-TCC TTC AAA CTA ATA AAA CTT TAC-3’ |  |
| mRNAstart | forward | 5’-TTT GTA AAG TTT TAT TAG TTT GAA GGA-3’ | 296 |
|  | reverse | 5’-GAC CCA ACC CAT TAA CCT C-3’ |  |
| Downstream | forward | 5’-CGA GGT TAA TGG GTT GGG T-3’ | 523 |
|  | reverse | 5’-CGC AAC TAA CAC AAA TTA AC-3’ |  |
